# Impact of the Common Genetic Associations of Age-Related Macular Degeneration upon Systemic Complement Component C3d Levels

**DOI:** 10.1371/journal.pone.0093459

**Published:** 2014-03-27

**Authors:** Tina Ristau, Constantin Paun, Lebriz Ersoy, Moritz Hahn, Yara Lechanteur, Carel Hoyng, Eiko K. de Jong, Mohamed R. Daha, Bernd Kirchhof, Anneke I. den Hollander, Sascha Fauser

**Affiliations:** 1 Department of Ophthalmology, University Hospital of Cologne, Cologne, Germany; 2 Department of Ophthalmology, Radboud University Nijmegen Medical Centre, Nijmegen, the Netherlands; 3 Institute of Medical Statistics, Informatics and Epidemiology, University of Cologne, Cologne, Germany; 4 Department of Nephrology, Leiden University Nijmegen Medical Center, Leiden, the Netherlands; Saitama Medical University, Japan

## Abstract

Age-related macular degeneration (AMD) is a common condition that leads to severe vision loss and dysregulation of the complement system is thought to be associated with the disease. To investigate associations of polymorphisms in AMD susceptibility genes with systemic complement activation, 2655 individuals were genotyped for 32 single nucleotide polymorphisms (SNPs) in or near 23 AMD associated risk genes. Component 3 (C3) and its catabolic fragment C3d were measured in serum and AMD staging was performed using multimodal imaging. The C3d/C3 ratio was calculated and associations with environmental factors, SNPs and various haplotypes of *complement factor H* (*CFH*) genes and *complement factor B (CFB*) genes were analyzed. Linear models were built to measure the influence of genetic variants on the C3d/C3 ratio. The study cohort included 1387 patients with AMD and 1268 controls. Higher C3d/C3 ratios were found for current smoker (p = 0.002), higher age (p = 1.56×10^−7^), AMD phenotype (p = 1.15×10^−11^) and the two SNPs in the C3 gene rs6795735 (p = 0.04) and rs2230199 (p = 0.04). Lower C3d/C3 ratios were found for diabetes (p = 2.87×10^−6^), higher body mass index (p = 1.00×10^−13^), the SNPs rs1410996 (p = 0.0001), rs800292 (p = 0.003), rs12144939 (p = 4.60×10^−6^) in *CFH*, rs4151667 (p = 1.01×10^−5^) in *CFB* and individual haplotypes in *CFH* and *CFB*. The linear model revealed a corrected R-square of 0.063 including age, smoking status, gender, and genetic polymorphisms explaining 6.3% of the C3d/C3 ratio. After adding the AMD status the corrected R-square was 0.067. In conclusion, none of the evaluated genetic polymorphisms showed an association with increased systemic complement activation apart from two SNPs in the C3 gene. Major genetic and non-genetic factors for AMD were not associated with systemic complement activation.

## Introduction

Age-related macular degeneration (AMD) is a neurodegenerative disease causing visual impairment and blindness in the elderly population. Accumulation of drusen between Bruch´s membrane and the retinal pigment epithelium characterizes the early forms while the two advanced forms show geographic atrophy and choroidal neovascularization. Risk is multifactorial including environmental and genetic factors. Genetic variation accounts for up to 71% of the disease risk [1]. Many genetic polymorphisms were found in the alternative pathway of the complement system including *complement factor H (CFH), complement component 3 (C3), Complement factor I (CFI)*, and *complement factor B (C2/CFB* locus) [2]–[8]. Complement proteins and their activation products have been identified in retinal deposits of AMD patients [3], [9]–[11].

The alternative complement pathway is constantly activated by the spontaneous hydrolysis of a thioester bond in C3 and a tight regulation including CFH is necessary to prevent excessive activation. It is hypothesized that a dysregulation of the complement system leads to tissue damage and finally AMD.

The dysregulation of the complement system or their activation fragments were also found systemically. In AMD patients, various components of the complement system were found at increased levels such as CFB, CFD, C3a, C5a, C3d, and Ba [12], [13].

While the association of genetic polymorphisms with AMD is well established, only polymorphisms in the C3 gene and few haplotypes in the CFH and CFB/C2 gene were found to be associated with complement activation products including factor C3d in two small cohorts [12], [13]. The impact of other AMD susceptibility genes on the regulation of systemic complement activation remains unclear. In our study, we analyzed the association of 32 single nucleotide polymorphisms (SNPs) in or near 23 AMD risk genes with the C3d/C3 ratio as a marker for chronic complement activation in a Caucasian cohort of 2655 participants.

## Methods

### Study population

2655 participants from the European Genetic Database (EUGENDA, www.eugenda.org) were included in the study. The study was performed in accordance with the tenets of the Declaration of Helsinki and the Medical Research Involving Human Subjects Act (WMO) and was approved by the local ethics committee of the University Hospitals in Cologne and Nijmegen. Written informed consent was obtained from all participants.

AMD staging was performed by grading of retinal images including stereo fundus photographs (FPs), fluorescein angiograms (FAs) and spectral domain optical coherence tomograms (SDOCTs) according to the standard protocol of the Cologne Image Reading Center (CIRCL) by certified graders (TR, LE). AMD was classified by the presence of pigmentary changes together with at least 10 small drusen (<63 μm) or the presence of intermediate (63–124 μm) or large drusen (≥125 μm diameter) in the Early Treatment Diabetic Maculopathy Study (ETDRS) grid or geographic atrophy and/or choroidal neovascularisation (CNV) secondary to AMD in at least one eye.

Demographic data and non-genetic parameters including history of smoking (current/past/never), regular alcohol intake (yes/no), body mass index (BMI), arterial hypertension (yes/no), diabetes (yes/no), rheumatoid arthritis (yes/no), thyroid disease (yes/no), kidney disease (yes/no) and history of allergy (yes/no) were obtained by standardized interviewer-assisted questionnaires.

### Complement component measurements and genetic analysis

Serum samples were used for C3d and C3 measurements. Serum was prepared by coagulation at room temperature. After centrifugation, the samples were stored at −80°C within 1 hour after collection. Complement component C3 and the activation fragment C3d were measured in serum samples as described previously [14]. The C3d/C3 ratio was calculated as a measure of C3 activation.

Genomic DNA was extracted from peripheral blood samples using standard procedures. Thirty-two SNPs in or near 23 AMD associated risk genes were chosen representing the majority of loci associated with AMD. Genotyping of SNPs in the *ARMS2* (rs10490924), *CFH* (rs1061170, rs800292, rs12144939, rs1410996), *CFI* (rs10033900, rs141853578), *C2* (rs9332739), *C3* (rs2230199, rs433594, rs6795735), *CFB* (rs4151667, rs641153), *CFD* (rs3826945), *LPL* (rs12678919), *LIPC* (rs10468017), *TIMP3* (rs9621532), *APOE2* (rs7412), *APOE4* (rs429358), *FADS1* (rs174547), *CETP* (rs2230199), *TLR* (rs4986790, rs3775291), *SERPING* (rs2511989), *ABCA4* (rs1800555, rs1800553, rs76157638), *VEGFA* (rs699946), *SPRYD7* (rs7995557), *COL8A1* (rs13081855), *COL10A1* (rs3812111), *SLC16A8* (rs8135665), *ADAMTS9-AS2* (rs6795735) genes were carried out as previously described [15].

### Haplotype analysis

In order to analyze the influence of haplotypes on C3d/C3 ratios, the posterior probability of each haplotype in the CFH gene including rs1061170, rs800292 and rs12144939 and in the CFB gene including rs4151667 and rs641153 was calculated using PHASE software, version 2.1 [16], [17].

### Statistical analysis

All calculations were performed using SPSS software version 21.0 (IBM Software and Systems, Armonk, NY, USA). C3d/C3 ratios are given as median and interquartile range (1^st^ quartile – 3^rd^ quartile). Due to the skewed nature of the data, the logarithm (log_10_) of the C3d/C3 ratios was used for analysis. Associations between logarithmic C3d/C3 ratios and genetic polymorphisms, haplotypes, phenotype and environmental factors were analyzed using t-tests or univariate analysis of variance (ANOVA) depending on number of variables. Associations between AMD phenotype and genetic polymorphisms were evaluated using logistic regression analysis. Linear models were performed to illustrate the influence of the genetic factors on complement activation. P-Values <0.05 were considered statistically significant.

## Results

### Demographics and non-genetic factors

Mean age of the study population was 73.2±8.0 years (75.8±8.1 years for AMD patients and 70.4±6.8 years for controls, p<0.001). Demographic data, phenotype and environmental factors are summarized in Table 1. C3d/C3 ratios showed significant differences for age with increasing levels (except the youngest group from 50–59 years) and phenotype with higher values for AMD patients. A significant association was also found for diabetes, smoking, and BMI.

**Table 1:** Median C3d/C3 ratios for non-genetic factors.

Non-genetic factor	N (%)	Median C3d/C3 ratio (IQR)	T-test/univariate ANOVA
Female sex	1547 (58.3)	0.00424 (0.00325–0.00561)	0.90
Male sex	1108 (41.7)	0.00433 (0.00328–0.00567)	
Age 50–59 years	61 (2.3)	0.00430 (0.00337–0.00598)	1.56×10^−7^
Age 60–69 years	879 (33.1)	0.00408 (0.00312–0.00550)	
Age 70–79 years	1140 (42.9)	0.00426 (0.00324–0.00547)	
Age 80–89 years	474 (17.9)	0.00462 (0.00356–0.00591)	
Age 90–99 years	97 (3.7)	0.00488 (0.00374–0.00705)	
No AMD	1268 (47.8)	0.00403 (0.00309–0.00536)	1.15×10^−11^
AMD	1387 (52.2)	0.00449 (0.00348–0.00586)	
No arterial hypertension	1600 (63.6)	0.00428 (0.00328–0.00563)	0.24
Arterial hypertension	917 (36.4)	0.00425 (0.00321–0.00556)	
No diabetes	2268 (90.8)	0.00430 (0.00330–0.00567)	2.87×10^−6^
Diabetes	231 (9.2)	0.00390 (0.00295–0.00495)	
No rheumatoid arthritis	2353 (93.5)	0.00426 (0.00327–0.00559)	0.35
Rheumatoid arthritis	164 (6.5)	0.00433 (0.00304–0.00564)	
No thyroid disease	2119 (84.2)	0.00426 (0.00326–0.00559)	0.77
Thyroid disease	398 (15.8)	0.00429 (0.00325–0.00559)	
No kidney disease	2403 (95.5)	0.00426 (0.00326–0.00559)	0.92
Kidney disease	114 (4.5)	0.00447 (0.00320–0.00572)	
No allergy	1984 (78.8)	0.00428 (0.00325–0.00562)	0.75
Allergy	533 (21.2)	0.00425 (0.00328–0.00552)	
Never smoker	1029 (43.0)	0.00431 (0.00325–0.00572)	0.002
Past smoker	1164 (48.6)	0.00415 (0.00320–0.00545)	
Current smoker	201 (8.4)	0.00451 (0.00355–0.00584)	
BMI <25	930 (40.1)	0.00464 (0.00360–0.00611)	1.00×10^−13^
BMI 25–29	1084 (46.7)	0.00408 (0.00312–0.00531)	
BMI ≥30	308 (13.3)	0.00376 (0.00287–0.00493)	

IQR  =  interquartile range (1^st^ quartile – 3^rd^ quartile).

### Associations of C3d/C3 levels with genetic polymorphisms

Significant associations of C3d/C3 levels were found in the *CFH* gene for the SNPs rs1410996, rs800292 and rs12144939, in the *CFB* gene for rs4151667 and in the *C3* gene for rs6795735 and rs2230199. In all SNPs of the *CFH* and *CFB* gene, these variants showed lower C3d/C3 ratios than the reference alleles, only variants in *C3* revealed higher values. After stratification in AMD cases and controls, associations for the major risk variants in rs1061170 (*CFHY402H*, p = 0.35 for no AMD; p = 0.55 for AMD) and rs10490924 (*ARMS2*, p = 0.75 for No AMD, p = 0.25 for AMD) with the C3d/C3 ratio could not be observed. A detailed analysis is outlined in Table 2.

**Table 2:** Median C3d/C3 ratios for single nucleotid polymorphisms (SNPs).

SNP	Homozygous non-variant (n)	Heterozygous variant (n)	Homozygous variant (n)	Median C3d/C3 ratio homozygous non-variant (IQR)	Median C3d/C3 ratio heterozygous variant (IQR)	Median C3d/C3 ratio homozygous variant (IQR)	Univariate ANOVA
ARMS2	GG = 1039	GT = 825	TT = 241	0.00425	0.00416	0.00446	0.31
rs10490924				(0.00338–0.00589)	(0.00320–0.00556)	(0.00336–0.00593)	
CFH	TT = 690	TC = 967	CC = 436	0.00425	0.00418	0.00433	0.66
rs1061170				(0.00320–0.00560)	(0.00323–0.00546)	(0.00335–0.00561)	
CFH	CC = 459	CT = 453	TT = 113	0.00436	0.00404	0.00355	0.0001
rs1410996				(0.00334–0.00570)	(0.00310–0.00529)	(0.00209–0.00491)	
CFH	GG = 1177	GA = 608	AA = 95	0.00427	0.00406	0.00394	0.003
rs800292				(0.00329–0.00567)	(0.00312–0.00535)	(0.00319–0.00516)	
CFH	GG = 1290	GT = 537	TT = 64	0.00435	0.00390	0.00373	4.60×10^−6^
rs12144939				(0.00329–0.00570)	(0.00305–0.00510)	(0.00287–0.00469)	
CFI	TT = 499	TC = 1056	CC = 547	0.00426	0.00420	0.00424	0.80
rs10033900				(0.00324–0.00570)	(0.003.24–0.00553)	(0.00325–0.00547)	
CFI	GG = 1862	GA = 10	AA = 0	0.00419	0.00485	N/A	0.18
rs141853578				(0.00321–0.00553)	(0.00415–0.00673)		
C2	CC = 927	CG = 70	GG = 0	0.00422	0.00362	N/A	0.05
rs9332739				(0.00320–0.00544)	(0.00299–0.00492)		
C3	CC = 1300	GC = 713	GG = 115	0.00415	0.00432	0.00432	0.04
rs2230199				(0.00319–0.00549)	(0.00334–0.00561)	(0.00327–0.00627)	
C3	CC = 723	CT = 900	TT = 261	0.00420	0.00420	0.00421	0.81
rs433594				(0.00320–0.00557)	(0.00323–0.00555)	(0.00328–0.00548)	
C3	GG = 1197	GA = 617	AA = 84	0.00413	0.00424	0.00448	0.04
rs6795735				(0.00317–0.00549)	(0.00334–0.00550)	(0.00333–0.00640)	
CFB	TT = 1958	TA = 160	AA = 2*	0.00428	0.00358	0.00497	1.01×10^−5^
rs4151667				(0.00327–0.00562)	(0.00297–0.00472)	(N/A)	
CFB	GG = 1616	GA = 265	AA = 7*	0.00421	0.00414	0.00311	0.69
rs641153				(0.00324–0.00556)	(0.00314–0.00559)	(0.00223–0.00335)	
CFD	TT = 488	TC = 407	CC = 93	0.00404	0.00421	0.00413	0.95
rs3826945				(0.00320–0.00542)	(0.00319–0.00531)	(0.00308–0.00525)	
LPL	AA = 1705	AG = 388	GG = 31	0.00423	0.00423	0.00479	0.89
rs12678919				(0.00321–0.00561)	(0.00338–0.00542)	(0.00342–0.00593)	
LIPC	CC = 1057	CT = 888	TT = 144	0.00421	0.00423	0.00441	0.46
rs10468017				(0.00322–0.00546)	(0.00322–0.00562)	(0.00337–0.00558)	
TIMP3	AA = 882	AC = 77	CC = 7*	0.00411	0.00425	0.00392	0.70
rs9621532				(0.00318–0.00531)	(0.00332–0.00543)	(0.00376–0.00584)	
APOE2	CC = 804	CT = 160	TT = 12	0.00420	0.00393	0.00417	0.53
rs7412				(0.00322–0.00543)	(0.00306–0.00524)	(0.00301–0.00485)	
APOE4	TT = 689	TC = 195	CC = 9*	0.00420	0.00396	0.00272	0.49
rs429358				(0.00320–0.00537)	(0.00311–0.00530)	(0.00253–0.00609)	
FADS1	TT = 995	TC = 924	CC = 209	0.00432	0.00416	0.00394	0.05
rs174547				(0.00330–0.00567)	(0.00319–0.00557)	(0.00334–0.00508)	
CETP	GG = 997	GT = 906	TT = 246	0.00420	0.00424	0.00428	0.91
rs2230199				(0.00328–0.00559)	(0.00320–0.00546)	(0.00324–0.00568)	
TLR	AA = 872	AG = 119	GG = 5	0.00419	0.00408	0.00283	0.24
rs4986790				(0.00319–0.00539)	(0.00333–0.00530)	(0.00248–0.00441)	
TLR3	CC = 497	CT = 380	TT = 86	0.00401	0.00420	0.00430	0.31
rs3775291				(0.00306–0.00561)	(0.00333–0.00513)	(0.00320–0.00594)	
SERPING	GG = 322	GA = 485	AA = 183	0.00410	0.00410	0.00428	0.56
rs2511989				(0.00317–0.00529)	(0.00322–0.00542)	(0.00316–0.00546)	
ABCA4	GG = 985	GA = 15	AA = 0	0.00418	0.00368	N/A	0.17
rs1800555				(0.00319–0.00538)	(0.00337–0.00440)		
ABCA4	GG = 975	GA = 4	AA = 0	0.00415	0.004.47	N/A	0.87
rs1800553				(0.00320–0.00538)	(0.00289–0.00659)		
ABCA4	GG = 1854	CG = 31	CC = 0	0.00419	0.00443	N/A	0.81
rs76157638				(0.00321–0.00556)	(0.00332–0.00521)		
VEGFA	AA = 629	AG = 322	GG = 42	0.00425	0.00400	0.00376	0.14
rs699946				(0.00327–0.00543)	(0.00303–0.00540)	(0.00324–0.00467)	
SPRYD7	TT = 714	TC = 220	CC = 21	0.00418	0.00433	0.00390	0.30
rs7995557				(0.00322–0.00537)	(0.00317–0.00558)	(0.00255–0.00503)	
COL8A1	GG = 1545	GT = 333	TT = 13	0.00421	0.00420	0.00502	0.73
rs13081855				(0.00322–0.00558)	(0.00321–0.00543)	(0.00342–0.00566)	
COL10A1	AA = 742	AT = 899	TT = 241	0.00424	0.00411	0.00440	0.87
rs3812111				(0.00319–0.00555)	(0.00323–0.00548)	(0.00330–0.00575)	
SLC16A8	CC = 1155	CT = 632	TT = 86	0.00421	0.00420	0.00423	0.83
rs8135665				(0.00324–0.00556)	(0.00319–0.00556)	(0.00312–0.00563)	
ADAMTS9	CC = 647	CT = 898	TT = 343	0.00425	0.00419	0.00412	0.57
rs6795735				(0.00320–0.00572)	(0.00325–0.00540)	(0.00313–0.00561)	

*Due to small number of cases excluded from univariate ANOVA analysis; IQR  =  interquartile range (1^st^ quartile – 3^rd^ quartile).

**Figure 1:**
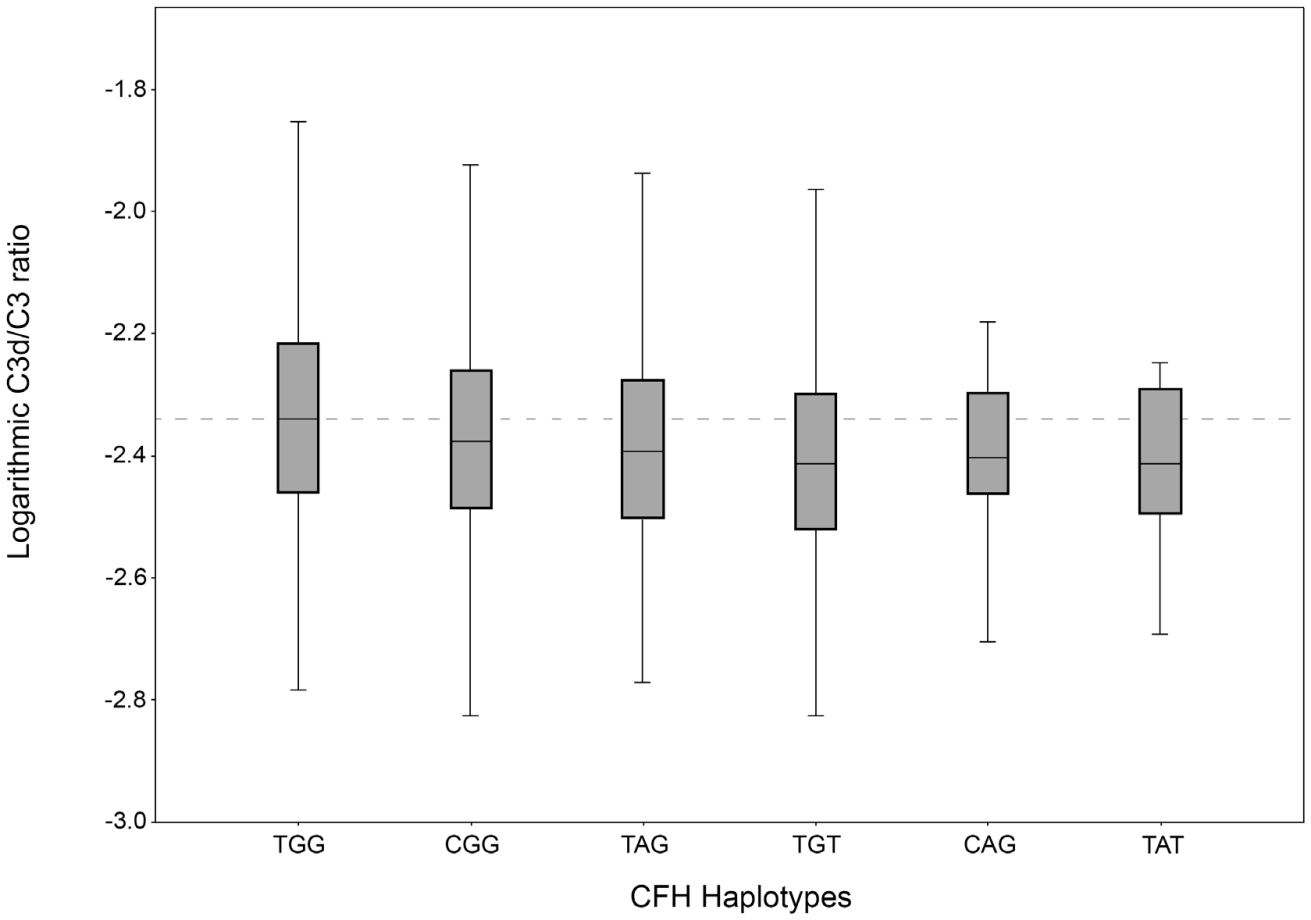
Logarithmic C3d/C3 ratios for haplotypes in the *CFH* gene rs1061170, rs800292 and rs12144939.

**Figure 2:**
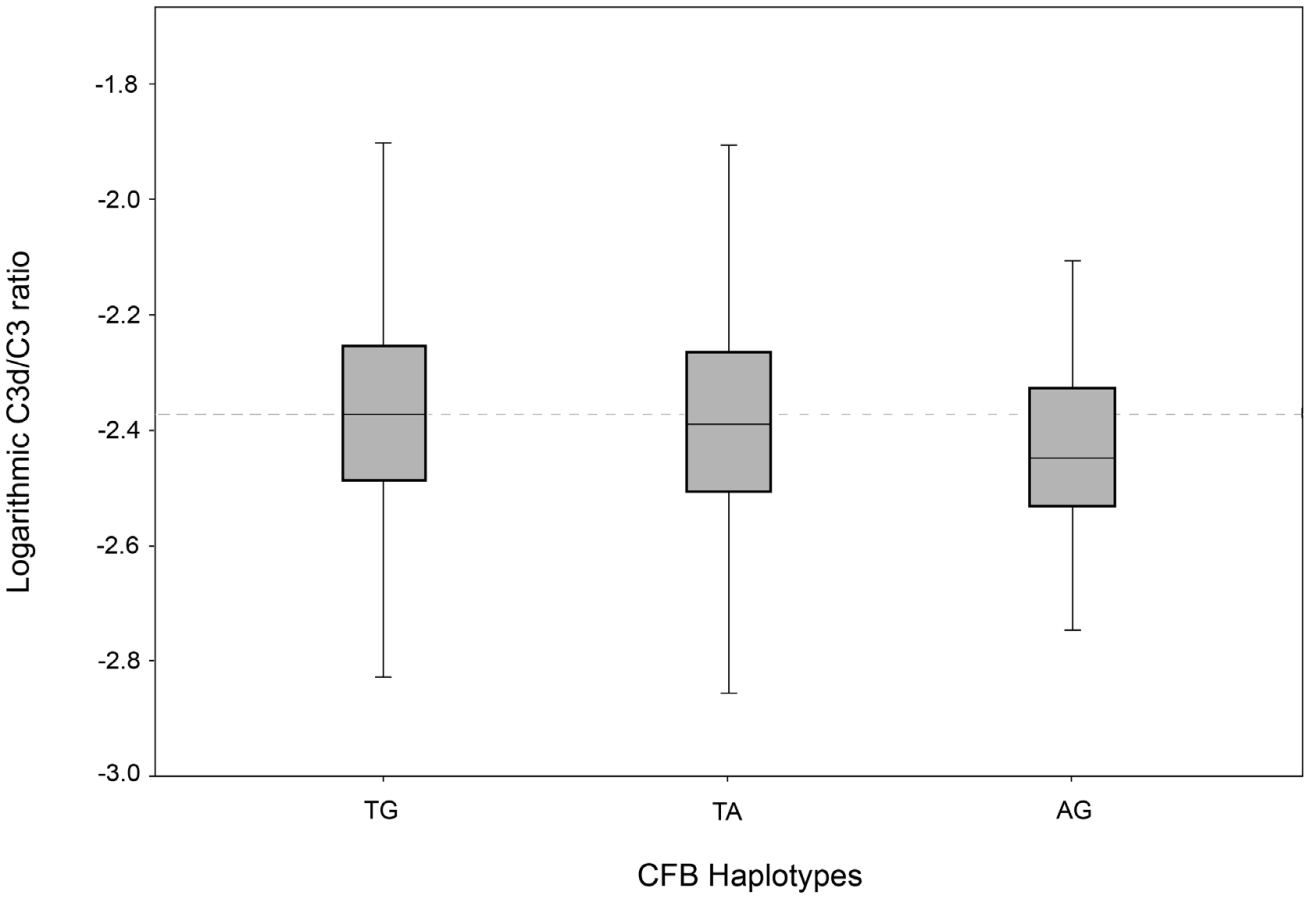
Logarithmic C3d/C3 ratios for haplotypes in the *CFB* gene rs4151667 and rs641153.

For the SNPs rs1061170, rs800292 and rs12144939 in the *CFH* gene and the SNPs rs4151667 and rs641153 in the *CFB* gene, haplotypes were associated with C3d/C3 levels (Table 3). All haplotypes were associated with lower C3d/C3 levels than the reference haplotype (Figure 1 and 2).

**Table 3:** Haplotypes for *CFH/CFB* and median C3d/C3 ratios.

Haplotype CFH	n	Haplotype frequency	MedianC3d/C3 ratio (IQR)	T-test
TGG	728	0.20	0.00456 (0.00345–0.00608)	-*
CGG	1544	0.42	0.00420 (0.00324–0.00550)	1.40×10^−8^
TAG	755	0.20	0.00405 (0.00314–0.00530)	3.61×10^−12^
TGT	620	0.17	0.00386 (0.00300–0.00504)	1.03×10^−13^
CAG	21	0.008	0.00396 (0.00339–0.00507)	-**
TAT	18	0.005	0.00386 (0.00317–0.00513)	-**
Haplotype CFB	n	Haplotype frequency	MedianC3d/C3 ratio (IQR)	T-test
TG	3337	0.89	0.00424 (0.00325–0.00559)	-*
TA	279	0.07	0.00408 (0.00310–0.00546)	0.15
AG	152	0.04	0.00356 (0.00292–0.00471)	9.96×10^−6^

For CFH single nucleotides polymorphisms rs1061170, rs800292, and rs12144939 and for CFB rs4151667 and rs641153 were chosen.

*T- test with comparison to reference haplotypes TGG and TG; **Due to small number of cases excluded from analysis; IQR  =  interquartile range (1^st^ quartile – 3^rd^ quartile).

### Associations of genetic polymorphisms with AMD

Performing logistic regression analysis, protective effects were found for variants in *CFH* rs1410996, *CFH* rs800292, *CFH* rs12144939, *CFB* rs641153 and *FADS1* rs174547. Variants in *CFB rs4151667, TIMP3 rs9621532 and APOE4 rs429358* showed a trend for a protective effect on AMD without reaching statistical significance, which may be due to low minor allele frequencies or smaller effects of those SNPs.

Variants in *ARMS2* rs10490924, *CFH* rs1061170, *C3* rs2230199, *C3* rs6795735 and *CETP* rs2230199 were found to be associated with significantly higher risk for AMD (Table 4). Variants in *VEGFA rs699946, SLC16A8 rs8135665 and ADAMTS9-AS2 rs6795735* also showed a trend for a higher AMD risk without statistical significance, which also may be due to a smaller effect on AMD development for each of those SNPs compared to *ARMS2 rs10490924* or *CFH rs1061170*.

**Table 4:** Logistic regression analysis between AMD and single nucleotide polymorphisms SNPs+.

SNP	Heterozygous variant			Homozygous variant		
	OR	95% CI	P-value	OR	95% CI	P-value
ARMS2 rs10490924	2.32	1.90–2.84	1.00×10^−13^	8.13	5.66–11.69	1.00×10^−13^
CFH rs1061170	1.57	1.27–1.95	3.16×10^−5^	3.70	2.82–4.85	1.00×10^−13^
CFH rs1410996	0.36	0.27–0.48	5.05×10^−12^	0.23	0.15–0.36	1.28×10^−13^
CFH rs800292	0.61	0.49–0.75	5.38×10^−6^	0.70	0.44–1.10	0.12
CFH rs12144939	0.57	0.47–0.71	6.71×10^−7^	0.51	0.28–0.91	0.02
CFI rs10033900	1.07	0.85–1.35	0.59	0.99	0.76–1.28	0.93
CFI rs141853578	1.17	0.96–1.43	0.12	-*	-*	-*
C2 rs9332739	0.70	0.42–1.17	0.17	-*	-*	-*
C3 rs2230199	1.17	0.96–1.42	0.11	2.17	1.42–3.31	0.0004
C3 rs433594	0.98	0.80–1.21	0.88	0.91	0.67–1.23	0.52
C3 rs6795735	1.10	0.89–1.36	0.37	2.06	1.27–3.33	0.03
CFB rs4151667	0.74	0.52–1.05	0.08	-*	-*	-*
CFB rs641153	0.72	0.54–0.96	0.02	-*	-*	-*
CFD rs3826945	1.01	0.76–1.34	0.94	0.76	0.48–1.22	0.26
LPL rs12678919	1.02	0.80–1.29	0.89	1.11	0.51–2.43	0.79
LIPC rs10468017	0.94	0.78–1.14	0.53	0.63	0.43–0.93	0.19
TIMP3 rs9621532	0.86	0.52–1.42	0.56	-*	-*	-*
APOE2 rs7412	1.21	0.84–1.76	0.31	-*	-*	-*
APOE4 rs429358	0.84	0.60–1.17	0.30	-*	-*	-*
FADS1 rs174547	0.88	0.72–1.06	0.18	0.64	0.46–0.88	0.006
CETP rs2230199	1.39	1.15–1.70	0.001	1.38	1.02–1.87	0.04
TLR rs4986790	1.08	0.71–1.64	0.71	0.53	0.08–3.36	0.50
TLR3 rs3775291	1.00	0.75–1.34	0.99	0.78	0.48–1.27	0.32
SERPING rs2511989	1.10	0.81–1.50	0.53	0.79	0.54–1.16	0.22
ABCA4D2177 rs1800555	0.96	0.33–2.82	0.94	-*	-*	-*
ABCA4G1961 rs1800553	0.88	0.12–6.58	0.90	-*	-*	-*
ABCA4 rs76157638	2.14	0.98–4.67	0.06	-*	-*	-*
VEGFA rs699946	1.08	0.81–1.44	0.61	1.47	0.75.2.89	0.27
SPRYD7 rs7995557	1.01	0.73–1.40	0.95	0.57	0.23–1.43	0.23
COL8A1 rs13081855	1.03	0.80–1.33	0.82	0.53	0.16–1.74	0.30
COL10A1 rs3812111	1.04	0.84–1.28	0.73	1.02	0.75–1.40	0.89
SLC16A8 rs8135665	1.21	0.98–1.49	0.08	1.39	0.87–2.21	0.17
ADAMTS9-AS2 rs6795735	1.21	0.98–1.51	0.08	1.30	0.98–1.72	0.07

+ Adjusted for age and gender; *analysis not performed due to small group size.

### Linear Models

Linear models were composed based on univariate analysis of covariance (ANCOVA) with the logarithmic C3d/C3 ratio as dependent variable to evaluate the influence of various factors on the C3d/C3 ratio. In the first model, we included all SNPs (rs1410996, rs800292, rs12144939 in *CFH*, rs4151667 in *CFB*, rs6795735 and rs2230199 in *C3*) that had reached statistical signifcance in the individual analysis and the two major AMD risk SNPs risk *ARMS2* rs10490924 and *CFH* rs1061170. Additionally, age, gender, and smoking status was included. The corrected R-square was 0.063. Adding the AMD status to the model, the corrected R-square was 0.067.

In the second model, *CFH* haplotypes, age, gender, and smoking status were included. The corrected R-square was 0.038.

## Discussion

Dysregulation of the alternative complement pathway is thought to play a key role in AMD pathogenesis, which is also reflected by increased systemic complement levels.

In this study we analyzed the association of genetic AMD risk polymorphisms with systemic complement activation. We identified only a few variants in the *CFH*, *CFB*, and *C3* gene that showed an association with systemic complement activation, while for all other genetic polymorphisms associations were not observed.

While the association with genetic polymorphisms was weak, we found a significant association of the phenotype AMD with an increased C3d/C3 ratio which is in line with other smaller studies.[12], [13] Our linear model including the AMD phenotype, the two major non-genetic risk factors age and smoking, and eight relevant SNPs could only explain 6.7% of the variation in the C3d/C3 ratio, indicating that these AMD risk polymorphisms do not explain sufficiently increased systemic complement activation found in AMD patients. The inclusion of *CFH* haplotypes in the model revealed an even lower explanation of the C3d/C3 ratio of only 3.8%.

Our analysis concentrated on C3d as a marker of chronic complement activation because it is a relatively stable protein with a long half-life. To correct for differences in the concentration of the precursor protein, we also measured C3 in the plasma.

CFH, CFB, and C3 influence the regulation of the alternative pathway of the complement system. CFH acts as the major regulator of complement activation controlling the alternative pathway in blood and on cell surfaces [18], and accelerates the decay of the alternative C3 convertase (C3bBb) [19].

CFH is also a cofactor of CFI-mediated cleavage and inactivation of C3b [20]. The formation of C3d, a polypeptide fragment generated during alternative C3 convertase cleaves C3 to C3b, is also CFH dependent [21]. Alterations in the CFH gene may change the regulating characteristics of CFH resulting in an up or down regulation of the CFH dependent elements of the alternative complement pathway. In our cohort, SNPs rs1410996, rs800292, and rs12144939 in the *CFH* gene were associated with lower C3d/C3 ratios and a lower risk for AMD, whereas the most common AMD risk variant rs1061170 was not associated with the C3d/C3 ratio even after stratification in AMD patients and controls. Additionally, *CFH* haplotypes showed lower C3d/C3 ratios in all cases compared to the reference haplotype. Therefore, *CFH* SNPs were not associated with increased systemic complement activation.

CFB is an acute phase protein involved in the alternative complement pathway as a precursor of C3 convertase. CFB is cleaved to Bb which combines with C3b to form the alternative pathway C3 convertase C3bBb. An acute phase response-mediated up-regulation may result in elevated systemic plasma levels of CFB in AMD patients and may contribute to an enhanced systemic complement activity [12], [13], [22]. In our study we observed lower C3d/C3 ratios for the operatively protective *CFB* variants for AMD indicating that individuals with these polymorphisms show less complement activation.

Among all analyzed SNPs, only variants in the *C3* gene were associated with higher systemic C3d/C3 ratios which aligns with the results by Hecker et al.^11^ The alternative pathway of the complement system starts with spontaneous hydrolysis of C3 and variants with a higher risk for AMD seem to influence this part of the complement cascade resulting in elevated systemic complement activation. Scholl et al did not observe a correlation between genetic variants in *C3* and systemic C3d levels^12^ underlining that the systemic effects of AMD susceptibility genes on complement activation are only weak.

In our study, slightly higher C3d/C3 levels were found in AMD patients. In order to not miss a combined effect of multiple SNPs, we performed linear models to illustrate the effect of the combination of SNPs on complement activation. These models could not explain the C3d/C3 ratio, showing that there have to be other systemic effects than AMD phenotype or genetic variants influencing systemic complement activation. Hecker et al also showed in a small cohort that risk haplotypes in CFH did not alter complement levels, whereas protective haplotypes reduced complement levels including C3d [12].

A limitation of our study is the analysis of only two components of the complement system, which is accompanied by several strengths including a large cohort of well-balanced AMD patients and controls, a high number of investigated SNPs and the use of multimodal imaging that avoids misclassification of phenotypes.

In summary, we showed that the major AMD risk polymorphisms in *CFH* and *ARMS2* are not associated with increased systemic complement activation as measured by the C3d/C3 ratio. Few SNPs were associated with lower levels of systemic complement activation, particularly the *CFH* and *CFB* polymorphisms that are protective against AMD. Only variants in *C3* were associated with elevated complement levels. Furthermore, a model including major genetic and non-genetic factors for AMD was not able to explain complement activation.
